# Astaxanthin Improves Stem Cell Potency via an Increase in the Proliferation of Neural Progenitor Cells

**DOI:** 10.3390/ijms11125109

**Published:** 2010-12-09

**Authors:** Jeong-Hwan Kim, Soo-Wan Nam, Byung-Woo Kim, Woobong Choi, Jong-Hwan Lee, Wun-Jae Kim, Yung-Hyun Choi

**Affiliations:** 1 Department of Biomaterial Control, Dong-Eui University, Busan, 614-714, Korea; E-Mails: 12845@deu.ac.kr (J.-H.K.); bwkim@deu.ac.kr (B.-W.K.); wbchoi@deu.ac.kr (W.C.); jonghwanlee@deu.ac.kr (J.-H.L.); 2 Department of Biotechnology and Bioengineering, Dong-Eui University, Busan 614-714, Korea; 3 Department of Blue-Bio Industry RIC, Dong-Eui University, Busan 614-714, Korea; 4 Department of Life Science & Biotechnology, Dong-Eui University, Busan 614-714, Korea; 5 Department of Urology, College of Medicine, Chungbuk National University, Cheongju, 361-763, Korea; E-Mail: wjkim@chungbuk.ac.kr (W.-J.K.); 6 Department of Biochemistry and Research Institute of Oriental Medicine, Dong-Eui University College of Oriental Medicine, Busan 614-052, Korea

**Keywords:** astaxanthin, neural progenitor cells, PI3K, proliferation, stem cell

## Abstract

The present study was designed to investigate the question of whether or not astaxanthin improves stem cell potency via an increase in proliferation of neural progenitor cells (NPCs). Treatment with astaxanthin significantly increased proliferation and colony formation of NPCs. For identification of possible activated signaling molecules involved in active cell proliferation occurring after astaxanthin treatment, total protein levels of several proliferation-related proteins, and expression levels of proliferation-related transcription factors, were assessed in NPCs. In Western blot analysis, astaxanthin induced significant activation of phosphatidylinositol 3-kinase (PI3K) and its downstream mediators in a time-dependent manner. Results of RT-PCR analysis showed upregulation of proliferation-related transcription factors and stemness genes. To estimate the relevance of PI3K and mitogen-activated protein, or extracellular signal-regulated kinase kinase (MEK) signaling pathways in cell growth of astaxanthin-treated NPCs, inhibition assays were performed with LY294002, a specific inhibitor of PI3K, and PD98059, a specific inhibitor of MEK, respectively. These results clearly showed that astaxanthin induces proliferation of NPCs via activation of the PI3K and MEK signaling pathways and improves stem cell potency via stemness acting signals.

## 1. Introduction

A stem cell is a special kind of cell, with a unique capacity to renew itself, to give rise to specialized cell types and for migration, proliferation, and cell survival [1]. Although most cells of the body, such as heart cells or skin cells, are committed to conduct specific functions, a stem cell is uncommitted and remains uncommitted until it receives a signal to develop into a specialized cell. Its proliferative capacity, combined with the ability to obtain active self renewal and to become specialized, ensures stem cells’ unique survival [1]. Neural progenitor cells (NPCs) have evoked great interest, given their expected capacity for self renewal resulting in expansion of the cell population. They also differentiate into desired cell types, thus representing new sources for cell replacement therapy [2,3]. To orchestrate self renewal via an increase in proliferation of NPCs, multiple signaling networks are activated by various intracellular and extracellular factors. Here, we illustrate the role of astaxanthin as an extracellular factor that induces improvement of self renewal via increased proliferation of NPCs.

Astaxanthin is widely distributed in nature, and is the principal pigment in crustaceans, salmonoids, and many other organisms [4–6]. It provides attractive pigmentation to many farm animals and also contributes to consumer appeal in the marketplace. In aquaculture, it is employed as a source of natural pigmentation and as a dietary supplement for trout and salmon [7,8]. This compound has important metabolic functions in animals, including neuroprotective actions [9–13], enhancement of immune response [14,15], and protection against diseases such as cancer [16,17] and antral ulcer [18,19] by scavenging of oxygen radicals. Antioxidant activity of astaxanthin has been reported to be approximately 10 times stronger than that of other carotenoids tested, including zeaxanthin, lutein, canthaxanthin, and β-carotene, and 100 times greater than that of α-tocopherol [20,21]. These effects are considered to be defense mechanisms against attack by reactive oxygen species. Astaxanthin also shows a strong activity as an inhibitor of oxygen radical-mediated lipid peroxidation [22,23] and inhibits hydrogen peroxide (H_2_O_2_)-mediated apoptotic cell death [24]. Therefore, astaxanthin has attracted commercial interest, not only as a pigmentation source, but also as a potent antioxidative reagent.

In the present study, we hypothesized that astaxanthin can improve stem cell potency via an increase in the proliferation potential of NPCs. To estimate this hypothesis, this study explored the ability of astaxanthin and signaling mechanisms that increase proliferation of NPCs.

## 2. Results and Discussion

### 2.1. Astaxanthin Induces Active Cell Proliferation and Improves Stem Cell Potency in NPCs via Stemness Acting Signals

**Figure 1.**
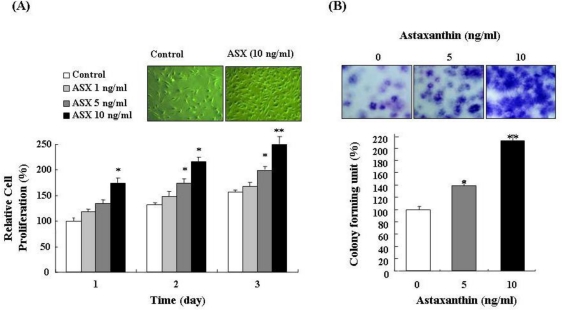
Astaxanthin stimulates cell proliferation of NPCs. (**A**) Proliferation of NPCs treated with different concentrations of astaxanthin (ASX) for 3 days were assessed by trypan blue exclusion. Application of astaxanthin for 3 days significantly increased proliferation of NPCs in a dose-dependent and time-dependent manner. Data were analyzed using analysis of variance with the Fisher test or *t*-test (* *P* < 0.05, ** *P* < 0.01); (**B**) A clonogenic (CFU) assay was performed to estimate proliferation efficiency of astaxanthin-treated NPCs. In the CFU assay, 10 ng/mL astaxanthin-treated NPCs showed an approximately 2-fold increase in colony formation compared with control NPCs. Data were analyzed using analysis of variance with the Fisher test or *t*-test (* *P* < 0.05, ** *P* < 0.01).

To confirm the effect of astaxanthin on cell growth, proliferation of NPCs treated with different concentrations (1, 5, and 10 ng/mL) of astaxanthin for three days was evaluated by trypan blue exclusion. As shown in Figure 1A, this treatment significantly increased proliferation of NPCs in a dose-dependent and time-dependent manner. In particular, 10 ng/mL astaxanthin showed the highest proliferation of NPCs. Therefore, 10 ng/mL was determined to be the optimal treatment for the study of NPCs. A clonogenic assay was also performed to estimate the proliferation efficiency of astaxanthin-treated NPCs. Because colony-forming units (CFU) are single cell populations, increases in CFU values show that astaxanthin can actively stimulate proliferation of NPCs. Proliferation efficiency of CFU in astaxanthin-treated cells was assessed via visual colony counts. In the CFU assay, astaxanthin-treated NPCs showed increased colony formation compared with control NPCs (Figure 1B). In particular, 10 ng/mL astaxanthin-treated NPCs showed an approximately two-fold increase in colony formation (Figure 1B).

**Figure 2.**
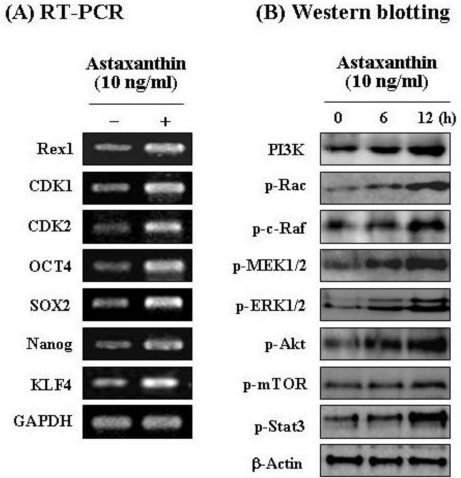
Astaxanthin induces active expression of several functional genes and stemness genes, and proliferation-related signal proteins in NPCs. (**A**) Astaxanthin induced upregulation of proliferation-related transcription factors (Rex1, CDK1, and CDK2), coupled with overexpression of stemness genes (OCT4, SOX2, Nanog, and KLF4); (**B**) Astaxanthin induced significant activation of PI3K and its downstream mediators, p-Rac, p-c-Raf, p-MEK, p-ERK, p-Akt, p-mTOR, and p-Stat3 in a time-dependent manner.

In both control NPCs and astaxanthin-treated cells, expression of molecular markers, including proliferation-related transcription factors and stemness genes, was assessed via RT-PCR. As shown in Figure 2A, 10 ng/mL astaxanthin applied for three days significantly induced upregulation of proliferation-related transcription factors (Rex1, CDK1, and CDK2), coupled with overexpression of stemness genes (OCT4, SOX2, Nanog, and KLF4) [25,26]. In particular, Rex1 expression was markedly increased in astaxanthin-treated cells. This result revealed that Rex1 expression is closely associated with proliferation of NPCs. In a recent study, we showed that Rex1 is a major gene, the expression of which is closely associated with proliferation of adipose tissue stromal cells [27]. Our present results are consistent with a recent report in which enhancement of Rex1 expression caused increased efficiency of cell proliferation. According to a recent report, four transcription factors (Oct4, Sox2, Klf4, and c-Myc) have been shown to reprogram primary mouse fibroblasts in culture [28]. Also, a balance between Klf4 and c-Myc is, in all likelihood, necessary for stable reprogramming in induced pluripotent stem cells [28]. In this study, astaxanthin-treated NPCs were shown to overexpress not only Oct4, Sox2, Nanog, and Rex1, but also Klf4 for the acquisition of active self-renewal activity (Figure 2A). Therefore, these results show that astaxanthin can induce active cell proliferation and that it improves stem cell potency in NPCs via stemness acting signals.

### 2.2. Astaxanthin Induces Proliferation of NPCs via PI3K and MEK Signaling Pathways

**Figure 3.**
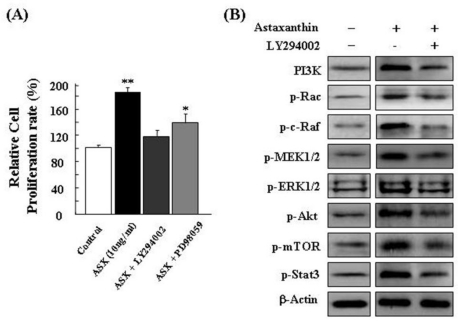

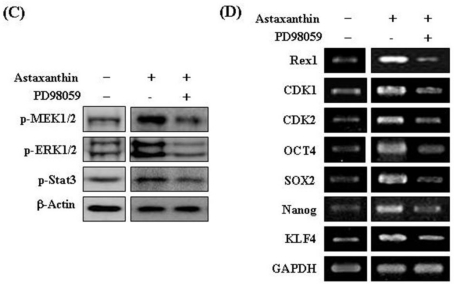
Astaxanthin induces proliferation of NPCs via the PI3K and MEK signaling pathways. To estimate the relevance of the PI3K and MEK signaling pathways in cell growth in astaxanthin-treated NPCs, inhibition assays were performed with LY294002 (10 μM) and PD98059 (10 μM). (**A**) Inhibition of PI3K and MEK has been shown to cause inhibition of cell growth in astaxanthin-treated NPCs *in vitro*; (**B**) LY294002 induced downregulation of PI3K, p-Rac, p-c-Raf, p-MEK, p-ERK, p-Akt, p-mTOR, and p-Stat3 proteins; (**C**) PD98059 significantly induced reduction of p-MEK, p-ERK, and p-Stat3; (**D**) PD98059 caused downregulation of proliferation-related transcription factors and stemness genes.

For identification of possible activated signaling molecules involved in active cell proliferation occurring after astaxanthin treatment, total protein levels of several proliferation-related proteins were assessed in NPCs by Western blot analysis. Figure 2B shows the results of Western blot in astaxanthin-treated NPCs for different lengths of time (0, 6, and 12 h). In Western blot analysis, astaxanthin induced significant activation of PI3K and its downstream mediators, p-Rac, p-c-Raf, p-MEK, p-ERK, p-Akt, p-mTOR, and p-Stat3 [29] in a time-dependent manner. This study then examined the relevance of the PI3K and MEK signaling pathways in cell growth in astaxanthin-treated NPCs. For these studies, inhibition assays were performed with specific inhibitors, LY294002 (a specific inhibitor of PI3K) and PD98059 (a specific inhibitor of MEK). Astaxanthin-treated NPCs were treated with LY294002 or PD98059, or were left untreated. After LY294002 and PD98059 treatment respectively, the relative cell proliferation rate of astaxanthin-treated NPCs was assessed by trypan blue exclusion: Results are shown in Figure 3A. PI3K and MEK inhibition have been shown to cause inhibition of cell growth in astaxanthin-treated NPCs *in vitro.* As shown in Figure 3B, the results of Western blot analysis indicated that LY294002 significantly induced downregulation of PI3K, p-Rac, p-c-Raf, p-MEK, p-ERK, p-Akt, p-mTOR, and p-Stat3 proteins.

In addition, PD98059 significantly induced reduction of p-MEK, p-ERK, and p-Stat3 proteins (Figure 3C). Results of RT-PCR also indicated that PD98059 caused downregulation of proliferation-related transcription factors (Rex1, CDK1, and CDK2) and stemness genes (OCT4, SOX2, Nanog, and KLF4) (Figure 3D). In this study, the activation of p-ERK in the astaxanthin-treated NPCs resulted in the induction of stemness transcription factor expression, particularly Rex1 expression (Figure 3). Conversely, inhibition of PI3K and MEK in NPCs induced a reduction in cell proliferation (Figure 3A) and stemness transcription factor expression (Figure 3D). Therefore, the upregulation of Rex1 production by astaxanthin is clearly required for cell proliferation. This result indicated that Rex1 is a major gene, the expression of which is closely associated with the proliferation of NPCs, and that astaxanthin increases NPCs proliferation efficiency via an enhancement of Rex1 expression. In conclusion, these results clearly showed that astaxanthin induces proliferation of NPCs via activation of the PI3K and MEK signaling pathways.

**Figure 4.**
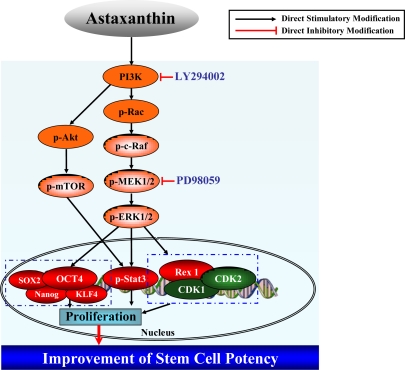
Putative cell proliferation mechanism induced by astaxanthin treatment. Astaxanthin induces proliferation of NPCs via activation of the PI3K and MEK signaling pathways.

Based on our present data, we suggest a model, shown in Figure 4, for explanation of the astaxanthin-induced cell proliferation mechanism through activation of the PI3K and MEK signaling pathways.

## 3. Experimental Section

### 3.1. Astaxanthin Reagent

Astaxanthin (3,3′-dihydroxy-β,β-carotene-4,4′-dione) was purchased from Sigma Chemicals (St. Louis, MO, U.S.) and used in all of the cell culture experiments. A stock solution of astaxanthin was made with dimethyl sulfoxide (DMSO) and stored at 4 °C. The stock solution was diluted to working concentrations prior to use.

### 3.2. Mouse Neural Progenitor Cells (NPCs) Culture

For preparation of NPCs, mice were anesthetized deeply using a pentobarbital in 0.9% sterile saline solution and sacrificed by decapitation. To obtain NPCs, the region of complete cervical enlargement (spinal cord level C3 through T1) was dissected out. The tissue was minced, washed in sterile Dulbecco’s phosphate buffered saline (DPBS), and digested in a solution of 0.125% of trypsin, DNase (0.01%, Sigma) in Hank’s balanced salt solution (HBSS) for 30 min at 37 °C. Cells were transferred to culture dishes containing serum free growth medium, which consists of neurobasal (NB) medium with B27 supplement, basic fibroblast growth factor (bFGF, 20 ng/mL), and epidermal growth factor (EGF, 20 ng/mL).

### 3.3. Selenium Treatment in NPCs and Analysis of Cell Viability

Cultured NPCs were seeded in 10 cm dishes at a density of 5 × 10^5^ and cultured in NB media at 37 °C in a CO_2_ incubator. Cells were then treated with astaxanthin at various concentrations (1, 5, and 10 ng/mL) for 3 days. Cell viability was assessed by visual cell counts in conjunction with trypan blue exclusion. In all viability assays, triplicate wells were used for each condition, and each experiment was repeated at least three times.

### 3.4. Colony-Forming Cell (CFU) Assay

Proliferation efficiency of colony-forming units (CFU) in astaxanthin-treated cells was assessed. Cultured NPCs were seeded in 10 cm dishes at a density of 5 × 10^5^ and cultured in NB media at 37 °C in a CO_2_ incubator. Cells were then treated with astaxanthin (10 ng/mL) for 3 days. For the CFU assay, control NPCs and astaxanthin-treated NPCs were seeded in 10-cm dishes at a density of 2 × 10^2^ and cultured in NB media at 37 °C in a CO_2_ incubator. After 15 days, cells were fixed with 4% paraformaldehyde (PFA) for 30 min at room temperature and stained with 0.1% toluidine blue in 1% PFA. Proliferation efficiency of the CFU was assessed via visual colony counts [30].

### 3.5. Western Blot Analysis

For confirmation of differentially expressed proteins after astaxanthin (10 ng/mL) treatment in cultured NPCs, NPCs and astaxanthin-treated cells were lysed in 500 μL of lysis buffer (20 mM Tris-HCl [pH 7.5], 150 mM NaCl, 1 mM EDTA, 1% Triton X-100, 2.5 mM sodium pyrophosphate, 1 mM EGTA, 1 mM glycerophosphate, 1 mM Na_3_VO_4_, and 1 mM PMSF. Lysates were clarified by centrifugation at 15,000 × g for 10 min, and the total protein content was determined using a Bio-Rad (Millan, Italy) protein assay kit. For Western blotting, equal amounts (40 μg) of protein extracts in a lysis buffer were subjected to 10% SDS-PAGE analysis and transferred to a nitrocellulose membrane [31]; anti-phosphatidylinositol 3-kinase (PI3K; 1:1000), anti-p-Rac (1:1000), anti-p-c-Raf (1:1000), anti-p-mitogen-activated protein or extracellular signal-regulated kinase kinase (MEK; 1:1000), anti-p-extracellular signal-regulated kinase (ERK; 1:1000), anti-p-Stat3 (1:1000), anti-p-Akt (1:1000), p-mTOR (Cell Signaling Technology, Danvers, MA, U.S.; 1:1000), and anti-β-Actin (Sigma) antibodies were then incubated with membranes. Relative band intensities were determined using Quality-one 1-D analysis software (Bio-Rad).

### 3.6. Reverse Transcription-Polymerase Chain Reaction (RT-PCR)

Total cellular RNA was extracted with Trizol (Life Technologies, Frederick, MD, U.S.). Concentration of RNA was determined spectrophotometrically, and concentration and integrity were further evaluated by agarose gel electrophoresis. Residual DNA, if any, was eliminated from the samples using DNase I (RNase-free, Takara Bio Inc, Kyoto, Japan). cDNA was synthesized from the RNA using an oligo-dT primer amplified by 35 cycles (94 °C, 1 min; 55 °C, 1 min; 72 °C, 1 min) of PCR using 20 pM of specific primers. PCR amplification was performed using the primer sets. Duplicate PCR reactions were amplified using primer designed glyceraldehyde-3-phosphate dehydrogenase (GAPDH) as a control for assessment of PCR efficiency and for subsequent analysis by 1.5% agarose gel electrophoresis. PCR products were stained with ethidium bromide [32].

### 3.7. Inhibition Assays

To confirm the relevance of the PI3K and MEK signaling pathways in controlling the growth of astaxanthin-treated NPCs, cells were seeded in 10 cm dishes at a density of 5 × 10^5^ and cultured in NB media at 37 °C in a CO_2_ incubator. Cells were then treated with astaxanthin (10 ng/mL) for 1 day. Astaxanthin-treated NPCs were treated with PI3K inhibitor LY294002 (10 μM; Promega, Madison, WI, U.S.), MEK inhibitor PD98059 (10 μM; Sigma), or were left untreated. Cells were analyzed by Western blot and RT-PCR [27].

### 3.8. Statistical Analysis

All data were presented as mean ± S.E.M from five or more independent experiments. Statistical significance of the differences between groups was calculated by using the Student’s two tailed *t*-test.

## 4. Conclusions

In the present study, astaxanthin significantly increased proliferation of NPCs in a dose-dependent and time-dependent manner. Also astaxanthin markedly induced upregulation of proliferation-related transcription factors (Rex1, CDK1, and CDK2), coupled with overexpression of stemness genes (OCT4, SOX2, Nanog, and KLF4) for the acquisition of active self-renewal activity. In particular, astaxanthin increased NPCs proliferation efficiency via an enhancement of Rex1 expression. Results of the CFU assay clearly indicated that astaxanthin can actively improve proliferation of NPCs, which was associated with significant activation of PI3K and its downstream mediators, p-Rac, p-c-Raf, p-MEK, p-ERK, p-Akt, p-mTOR, and p-Stat3 in a time-dependent manner. Results from inhibition assays clearly showed that astaxanthin effectively induces proliferation of NPCs via activation of the PI3K and MEK signaling pathways. In conclusion, our data indicated that astaxanthin can improve stem cell potency via increased proliferation of NPCs.
